# Resolving spatial response heterogeneity in glioblastoma

**DOI:** 10.1007/s00259-024-06782-y

**Published:** 2024-06-05

**Authors:** Julian Ziegenfeuter, Claire Delbridge, Denise Bernhardt, Jens Gempt, Friederike Schmidt-Graf, Dennis Hedderich, Michael Griessmair, Marie Thomas, Hanno S Meyer, Claus Zimmer, Bernhard Meyer, Stephanie E Combs, Igor Yakushev, Marie-Christin Metz, Benedikt Wiestler

**Affiliations:** 1 Department of Neuroradiology, School of Medicine and Health, Technical University of Munich, 81675 München, Germany; 2 Department of Pathology, Technical University of Munich, 81675 München, Germany; 3 Department of Radiation Oncology, School of Medicine and Health, Technical University of Munich, 81675 München, Germany; 4 Department of Neurosurgery, School of Medicine and Health, Technical University of Munich, 81675 München, Germany; 5 Department of Neurosurgery, University Medical Center Hamburg-Eppendorf, 20251 Hamburg, Germany; 6 Department of Neurology, School of Medicine and Health, Technical University of Munich, 81675 München, Germany; 7 Department of Nuclear Medicine, School of Medicine and Health, Technical University of Munich, 81675 München, Germany; 8 TranslaTUM, Technical University of Munich, 81675 München, Germany

**Keywords:** Glioblastoma, PET, DSC perfusion, Pseudoprogression, Spatial heterogeneity, Radiomics

## Abstract

**Purpose:**

Spatial intratumoral heterogeneity poses a significant challenge for accurate response assessment in glioblastoma. Multimodal imaging coupled with advanced image analysis has the potential to unravel this response heterogeneity.

**Methods:**

Based on automated tumor segmentation and longitudinal registration with follow-up imaging, we categorized contrast-enhancing voxels of 61 patients with suspected recurrence of glioblastoma into either true tumor progression (TP) or pseudoprogression (PsP). To allow the unbiased analysis of semantically related image regions, adjacent voxels with similar values of cerebral blood volume (CBV), FET-PET, and contrast-enhanced T1w were automatically grouped into supervoxels. We then extracted first-order statistics as well as texture features from each supervoxel. With these features, a Random Forest classifier was trained and validated employing a 10-fold cross-validation scheme. For model evaluation, the area under the receiver operating curve, as well as classification performance metrics were calculated.

**Results:**

Our image analysis pipeline enabled reliable spatial assessment of tumor response. The predictive model reached an accuracy of 80.0% and a macro-weighted AUC of 0.875, which takes class imbalance into account, in the hold-out samples from cross-validation on supervoxel level. Analysis of feature importances confirmed the significant role of FET-PET-derived features. Accordingly, TP- and PsP-labeled supervoxels differed significantly in their 10th and 90th percentile, as well as the median of tumor-to-background normalized FET-PET. However, CBV- and T1c-related features also relevantly contributed to the model’s performance.

**Conclusion:**

Disentangling the intratumoral heterogeneity in glioblastoma holds immense promise for advancing precise local response evaluation and thereby also informing more personalized and localized treatment strategies in the future.

**Supplementary Information:**

The online version contains supplementary material available at 10.1007/s00259-024-06782-y.

## Introduction

A significant challenge in the management of glioblastoma patients is distinguishing between true tumor progression (TP) and pseudoprogression (PsP) in the disease course [1]. TP and PsP often present similarly on conventional MRI scans, displaying mass effect, edema, and contrast enhancement. Accurately determining whether a patient is experiencing TP or PsP is clinically crucial, as these conditions necessitate distinct treatment approaches and have different prognoses [2]. 

While conventional contrast-enhanced MRI struggles to differentiate between TP and PsP, advanced imaging techniques such as perfusion-weighted imaging (PWI) and amino acid positron emission tomography (PET) offer insights into tumor physiology and key oncogenic processes. They thereby provide an essential spectrum of physiological and biophysical imaging features to improve diagnostic accuracy in this challenging situation [3]. ^,^ [4]^,^ [5] Dynamic susceptibility contrast perfusion imaging (DSC-PWI) is a T2*-weighted MRI sequence that relies on the injection of a gadolinium-based contrast agent to measure brain perfusion and estimate tumor blood volume, a marker of angiogenesis [6]. PET scans using O-(2-[18 F]fluoroethyl)-L-tyrosine (FET-PET) tracer detect amino acid uptake in cancer cells, depicting tumor cell metabolism [7]. ^,^ [8].

Multiple studies suggest that using a combination of imaging modalities leads to better results in distinguishing between TP and Psp than using a single modality alone [9]. However, effectively integrating data from different techniques and modalities remains challenging.

The morphological hallmark of glioblastoma is its heterogeneity, which is evident across various aspects of tumor biology [10]. Each glioblastoma consists of different subclones that differ in their genomic, cellular, and functional characteristics [11]. This variability likely leads to differences in response to therapy and is probably at the root of treatment failure. This spatial intratumoral heterogeneity poses an additional challenge in therapy monitoring for patients with suspected tumor recurrence. Histopathologic samples indicate that clear differentiation between TP and PsP is often unfeasible, even with multiple biopsies, as mixed areas of both TP and PsP are common [12]. ^,^ [13].

To date, however, virtually all studies looking into the differentiation of TP and PsP assume a “black and white” differentiation, categorizing the entire scan as either TP or PsP while neglecting the intratumoral response heterogeneity [14]. ^,^ [15]^,^ [16] Clearly, advanced, non-invasive tools are needed to visualize spatial heterogeneity and to monitor treatment response regionally. These tools may complement or serve as alternatives to biopsy, aiding in informed decision-making.

To address this, we aim to develop an automated pipeline that allows us to analyze tumor response over space and time and build a predictive model for regional tumor response from rich, multimodal imaging data. This innovative approach holds the potential to dissect intratumoral heterogeneity, deepen our understanding of therapy response and resistance, and inform personalized treatment strategies for glioblastoma patients.

## Methods and materials

### Patient data

This retrospective study was approved by our local Institutional Review Board (# 340/16S). We included patients presenting with suspected tumor recurrence (per discussion in our interdisciplinary neurooncology tumor board) of histologically confirmed glioblastoma (*isocitrate dehydrogenase* (*IDH*) wild-type, classified as WHO-CNS grade 4 according to the 2021 WHO classification of CNS tumors [17]) between January 2016 and November 2021. All patients had initially undergone maximal safe resection, followed by radiochemotherapy (Stupp protocol) [18]. To be included, we required the availability of an advanced MRI protocol (including DSC perfusion raw data) as well as a FET-PET scan at the time of suspected progression (with a maximum interval of four weeks between PET and MRI) after radiotherapy [19]. We consider this scan the baseline. Further, to assess spatial heterogeneity and differentiate between areas of true tumor progression and pseudoprogression, we required a follow-up MRI at a maximal 16-week interval, without initiation of a salvage therapy in-between. In the follow-up protocol, structural MRI sequences were sufficient. Imaging evaluations conformed to the established Modified Response Assessment in Neuro-Oncology (mRANO) criteria [20]. 

### Image acquisition

In our study, magnetic resonance (MR) imaging was primarily performed using a Philips 3 Tesla whole-body scanner (*n* = 63; Achieva or Ingenia, Philips, Best, The Netherlands) or a Siemens Verio 3 Tesla whole-body scanner (*n* = 3; Siemens, Erlangen, Germany).

The MR imaging protocols included an isotropic fluid-attenuated inversion recovery (FLAIR) sequence with an isotropic voxel size of 1 mm³ and an isotropic T1-weighted Turbo Field Echo (TFE) sequence with a voxel size of 1 mm³, acquired both pre- and post-contrast administration. Additionally, an axial T2-weighted sequence with a voxel resolution of 0.36 × 0.36 × 4 mm and dynamic susceptibility contrast (DSC) perfusion imaging with a voxel size of 1.75 × 1.75 × 4 mm were conducted.

The FET-PET imaging was acquired using a PET/MR scanner (Biograph mMR, Siemens Healthcare GmbH, Erlangen, Germany) (*n* = 55) or a PET/CT (Biograph mCT; Siemens Healthcare, Knoxville, TN, USA) (*n* = 11), adhering to an established clinical protocol. Patients were instructed to fast for at least four hours prior to the scan. Emission images were captured between 30 and 40 min subsequent to the intravenous administration of a specified dosage of 185 ± 10% MBq of 18 F-FET. Attenuation correction was performed according to the vendor’s protocol.

### Image processing of the baseline

CBV (cerebral blood volume) maps were calculated using a leakage-correction algorithm from the raw DSC perfusion data [21]. Next, all images and parameter maps (including the attenuation-corrected FET-PET image and the CBV maps) from the baseline imaging were rigidly co-registered into the SRI24 atlas space using NiftyReg and skullstripped using HD-BET [22]. ^,^ [23] We automatically segmented tumor subregions into contrast-enhancing tumor (CET), necrosis, and edema by applying the freely available BraTS.Toolkit, which ensembles various state-of-the-art image segmentation algorithms [24]. In case of missing T2w or FLAIR images, those sequences were synthesized using a GAN-based approach to improve automated segmentation [25]. In addition, tissue maps (gray matter, white matter, CSF) were automatically generated using ANTs Atropos [26]. From the resulting white matter tissue maps, automated tumor-to-background normalization of the FET-PET data was performed. All image registrations and segmentations were checked and corrected when necessary using ITK-SNAP [27]. 

### Response heterogeneity assessment

**Fig. 1:**
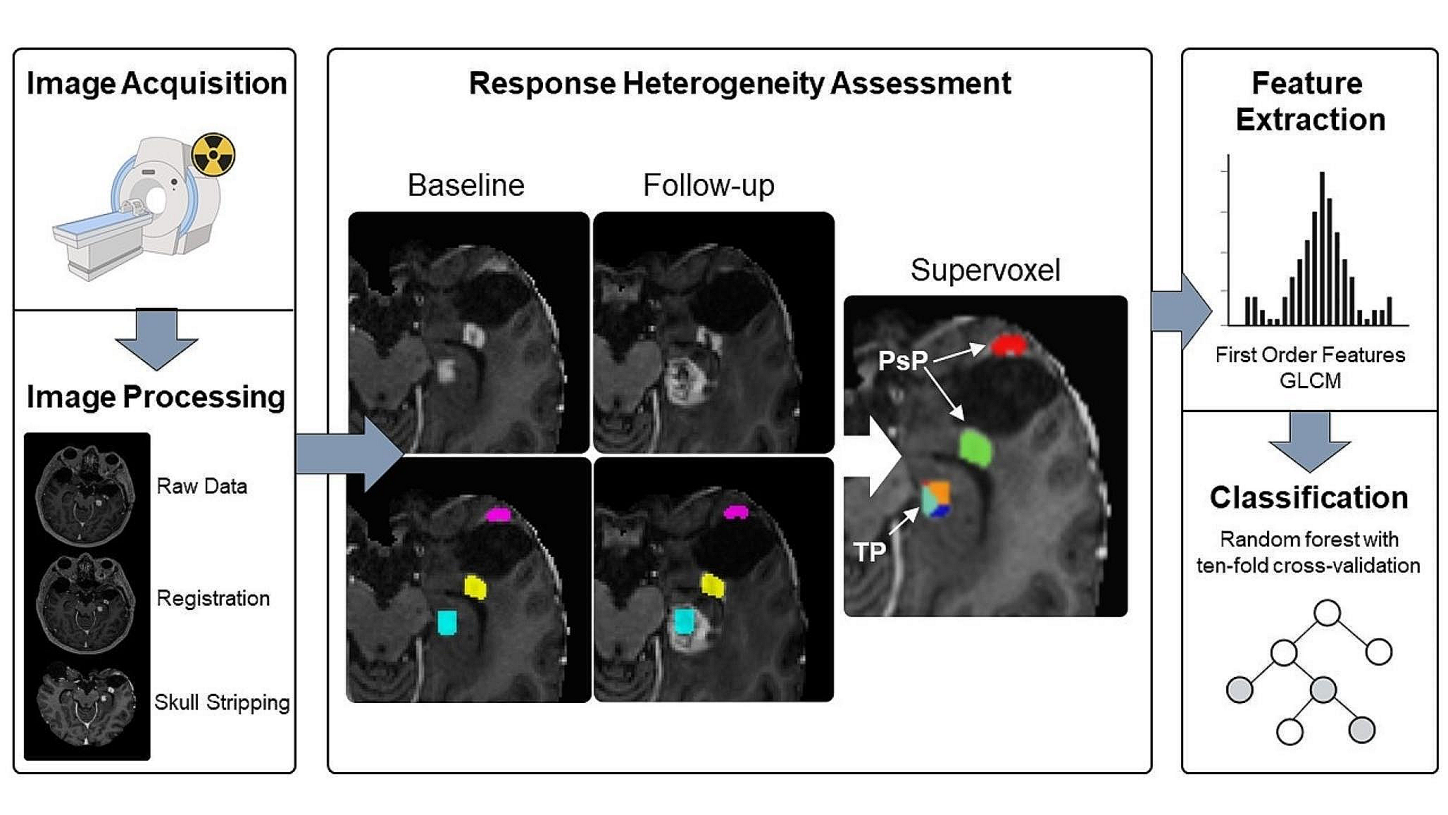
Study workflow. After image processing, including longitudinal registration, contrast-enhancing areas are labeled as either true tumor progression (TP) when they show further progression in follow-up imaging (turquoise color) or pseudoprogression (PsP) (decreasing contrast enhancement = pink, stable enhancement = yellow) to allow response heterogeneity assessment. Next, these contrast-enhancing areas are grouped into supervoxel areas, from which first-order and texture features (GLCM = Gray Level Co-Occurrence Matrix) are extracted to train a Random Forest classifier for response prediction in a tenfold cross-validation scheme. In the illustrative image with the heading “supervoxel”, each color represents one supervoxel

To establish a ground truth in spatial response heterogeneity assessment by differentiating between areas of true tumor progression and pseudoprogression, we co-registered the contrast-enhanced T1w scan from the follow-up MRI onto the baseline MRI. For this, we performed a linear registration (using NiftyReg), as we found this to produce accurate alignment given the relatively short interval between scans while avoiding a potential deformation of tumors (which might obscure the response assessment) from nonlinear registration. This way, areas of further progressive contrast-enhancement in the follow-up scan, indicating vital, progressive tumor areas (TP), could be distinguished from areas of stable or decreasing contrast-enhancement (indicating PsP) and labeled manually by J.Z. as a ground truth. Subsequently, these areas could be mapped back to the contrast enhancement in the baseline image. For this assessment, we relied on the mRANO criteria [20]. This is illustrated in Fig. 1.

### Radiomics model building

To facilitate regional prediction of response in the baseline image, we performed a supervoxel segmentation of a multi-channel input (CBV, FET-PET, contrast-enhanced T1w) in the areas of contrast enhancement [28]. A supervoxel is a 3D region that groups together similar adjacent voxels in an image to allow analysis of semantically similar image regions. Here, we used the “slic” implementation from scikit-image. Slic uses k-means clustering to arrange neighboring voxels based on their similarity in CBV, FET-PET, and contrast-enhanced T1w sequences to subdivide the contrast-enhancing tumor into smaller, (relatively) homogenous areas. We set the (rough) supervoxel size to 200 voxels to balance size and assessment granularity. Each supervoxel was assigned the follow-up label (TP or PsP) the majority of its voxels belonged to. Next, we employed pyradiomics to extract first-order statistics as well as gray-level co-occurrence matrix texture features [29]. Following the IBSI recommendations, this resulted in 126 features per supervoxel, 42 each from CBV, FET-PET, and contrast-enhanced T1w images [29, 30]. 

To train a predictive model to distinguish areas of later TP and PsP, we trained a Random Forest classifier [31]. A Random Forest is a versatile machine-learning algorithm that creates multiple weak, but uncorrelated decision trees and combines their predictions to make accurate classifications. We trained 100 decision trees using an entropy-based split criterion. Training and validation followed a tenfold cross-validation scheme, where we stratified the fold selection by patient, i.e., we made sure that all supervoxels from one patient were contained in the same fold to avoid information leakage. In addition, we extracted the impurity-based feature importances.

### Statistical analysis

All analyses were carried out in Python 3.8 and utilized the open-source libraries scikit-image 0.21 and scikit-learn 1.2.2 for image processing and machine learning, respectively, and pyradiomics 3.1.0 for feature extraction. We calculated the area under the curve statistics from the tenfold cross-validated prediction probabilities. Using a pre-defined probability cutoff of 0.5, we further binarized these probabilities into cross-validated predictions to calculate performance metrics, including (balanced) accuracy, sensitivity, and specificity. To compare features between both groups, we employed a non-parametric Mann-Whitney-U test.

## Results

### Patient cohort

The study included 61 patients (66 cases) who met the inclusion criteria. For the five patients included twice, at least one salvage therapy (usually re-irradiation) was performed between the first and second inclusion. Table 1 lists relevant patient characteristics. The mean age of the cohort was 59.6 years (standard deviation (SD) = 10.7). 36 patients (59%) were male and 25 (41%) were female. The median interval between the MRI and PET scans was 7 days (interquartile range (IQR) = 0–13), and the median time between the baseline and the follow-up MRI was 65.5 days (IQR: 40-95.75).

Of the 66 cases in our study, 37 (56%) were classified as TP, 12 (18%) as PsP, and 17 (26%) as mixed areas (coexistence of TP and PsP) based on expert consensus at follow-up imaging and according to mRANO criteria.

Of the 33 cases with MGMT methylation, 15 (45%) were classified as TP, 7 (21%) as Psp, and 11 (33%) as mixed. Of the 21 patients without MGMT methylation, 21 (72%) were classified as TP, 4 (14%) as PsP, and 4 (14%) as mixed. The higher likelihood of TP in MGMT unmethylated tumors (72% vs. 45% in MGMT methylated) supports previous findings [32]. ^,^ [33].

**Table 1:** Patient and tumor characteristics

Patient and tumor characteristics		
**Sex m/f**	36 (59%)	25 (41%)
**Age (mean; SD in years)**	59,8	± 10,7
**Interval between MRI and PET in days (median; IQR in days)**	7	0–13
**Interval between MRI BL and FU in days (median; IQR in days)**	65.5	40-95.75
**Supervoxel classification**		
TP	1069	49%
Psp	1128	51%
Total	2197	
**Supervoxels (median; IQR)**	24	6–67
**Expert consensus in FU**		
TP	37	56%
Psp	12	18%
Mixed areas	17	26%
Total	66	
**MGMT**		
Methylated	33	50%
Unmethylated	29	44%
N/A	4	6%
Total	66	
**MGMT Methylated**		
TP	15	45%
PsP	7	21%
Mixed areas	11	33%
Total	33	
**MGMT Unmethylated**		
TP	21	72%
PsP	4	14%
Mixed areas	4	14%
Total	29	

BL = baseline MRI, FU = follow-up MRI, TP = true tumor progression, PsP = pseudoprogression, MGMT = O-6-Methylguanin-DNA-Methyltransferase, N/A = not available

### Model performance

**Fig. 2:**
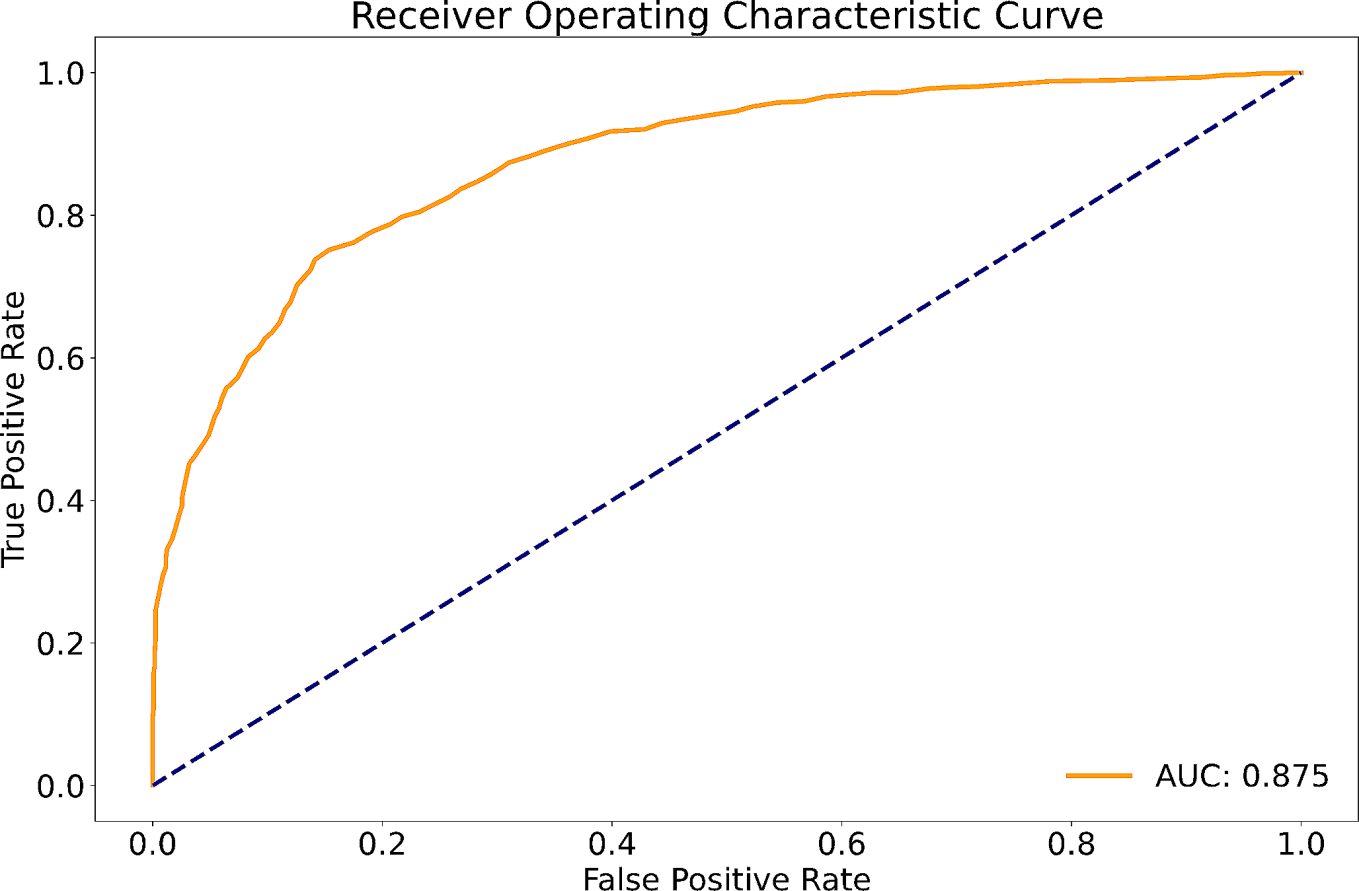
Receiver Operating Characteristic (ROC) curves of the Random Forest classifier (RF) to distinguish areas of later TP and PsP in the hold-out data from tenfold cross-validation

Supervoxel segmentation generated a total number of 2197 supervoxels (IQR: 6–67). Across all supervoxels, the distribution between areas labeled TP or PsP was relatively even, with 49% of the supervoxels classified as TP (*n* = 1069) and 51% as Psp (*n* = 1128). Receiver operating characteristic (ROC) analysis to identify TP versus PsP in a supervoxel demonstrated robust diagnostic performance. With results obtained from a tenfold cross-validation approach, the model achieved a macro-weighted AUC of 0.875, as shown in Fig. 2, accompanied by a high accuracy rate of 80% in the hold-out data. The individual AUCs in the ten folds ranged from 0.838 to 0.903, with no indication of a systematic bias (Supplementary Table 1 details the individual AUC per run).

To evaluate the effectiveness of our model, we calculated key performance metrics for distinguishing TP from PsP in glioblastoma. For TP, the model demonstrated a precision of 0.82, a recall of 0.75, and an F1 score of 0.78. In the case of PsP, the precision was 0.78, the recall was 0.84, and the F1 score was 0.81.

### Feature importances

**Fig. 3:**
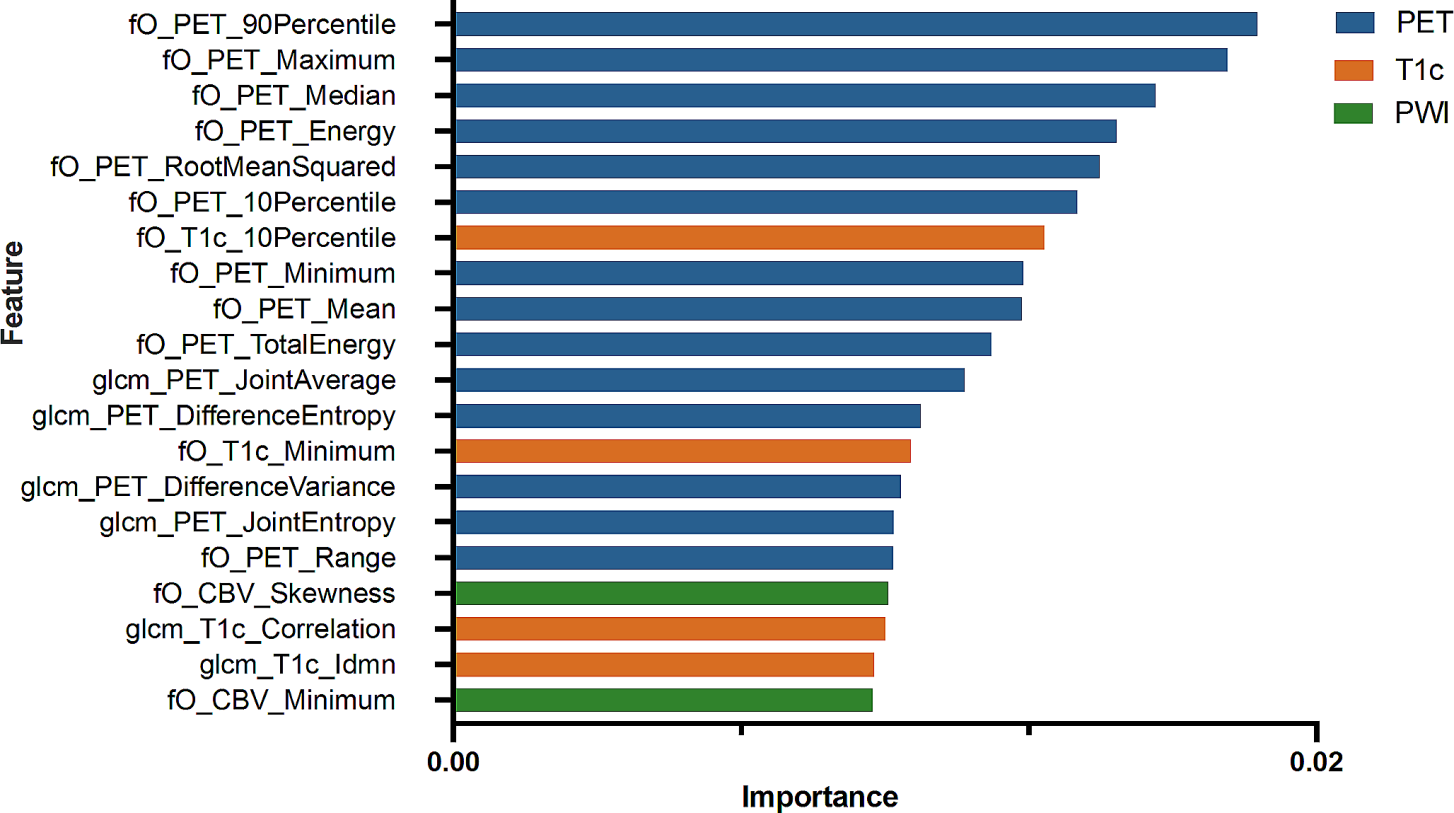
Ranking of top 20 feature importance. The length of each bar represents the relative importance (which sums to 1 over all features) of each input feature on the classifier’s performance. Note that a total of 126 features were extracted from each supervoxel, and only the top 20 are shown here

After reviewing the final model and analyzing the specific contributions of imaging modalities as shown in Fig. 3, we found that features from FET-PET played the most significant role in the classifier’s performance, making up 14 of the top 20 features. In addition, features obtained from conventional MRI and CBV maps were also influential, highlighting the reliance of the classifier on multimodal imaging.

**Fig. 4:**
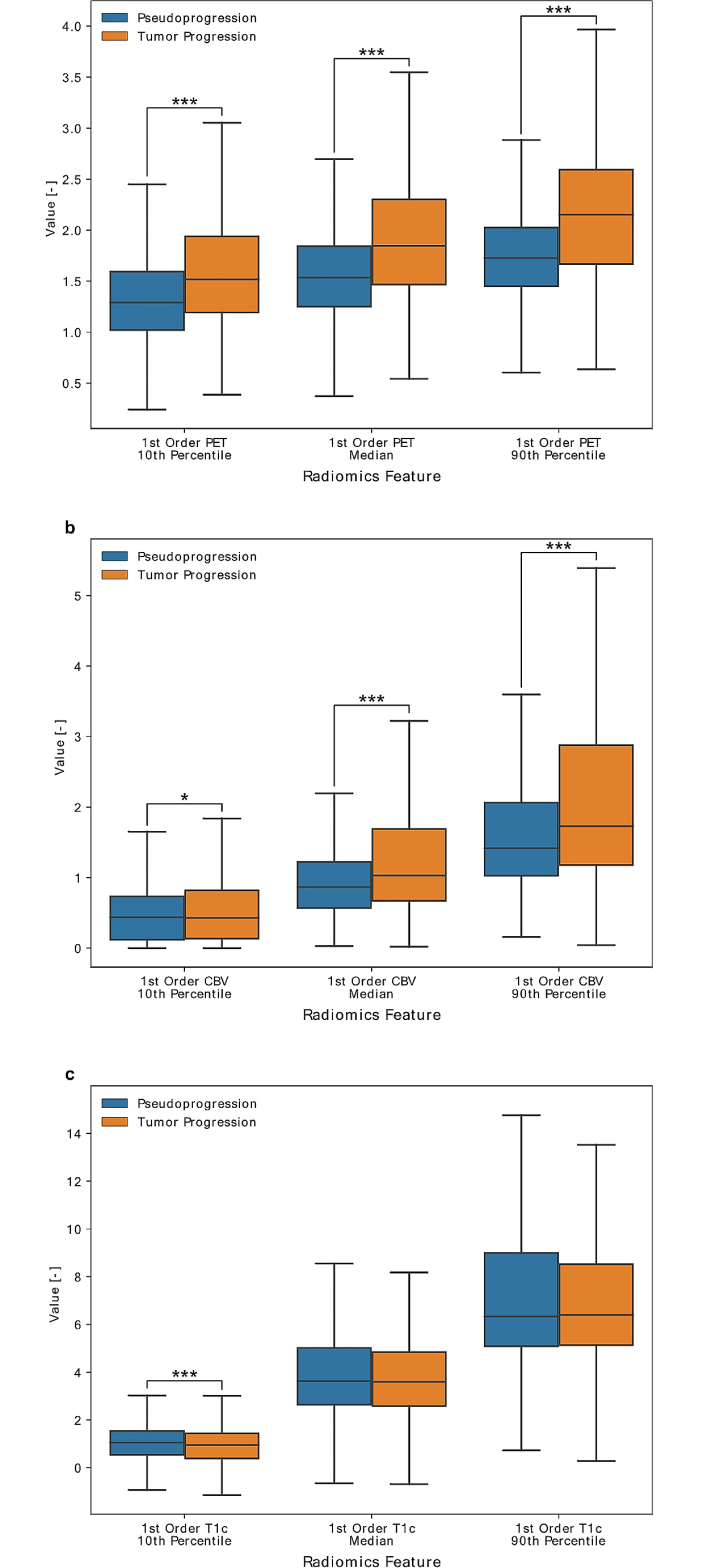
Comparison of tangible features between TP and PsP areas. Boxplots display the distribution of absolute values of **a** PET-, **b** CBV- and **c** T1c-derived features based on n = 2197 supervoxels labeled as PsP (blue) and TP (orange), respectively. Asterisks indicate significance levels, i.e., *p* < 0.05 (*), *p* < 0.01 (**), and *p* < 0.001 (***)

Looking into the actual values of each modality’s features, we indeed found significant differences between TP and PsP areas of the tumors in 10th and 90th percentile, as well as in the median values of FET-PET uptake (*p* < 0.001, Mann-Whitney-U test, Fig. 4a, Supplementary Table 2).

Also, CBV values differed significantly in their 10th and 90th percentile (*p* < 0.05, Mann-Whitney-U test, Fig. 4b, Supplementary Table 2).

In contrast to that, T1C-values were different on a significant level only in their 10th percentile (*p* < 0.001, Mann-Whitney-U test, Fig. 4c, Supplementary Table 2).

## Discussion

Differentiating between TP and PsP remains a significant clinical challenge in the management of glioblastoma patients. In reality, the response of a tumor to therapy typically will be mixed (i.e., areas of progressive tumor and stable disease co-exist in one patient). Presently, clinical practice tends to oversimplify the spatially heterogeneous response by making a global evaluation, disregarding this complexity. However, a more nuanced assessment holds promise for guiding local therapies like resection or targeted (re-)irradiation.

To address this challenge, we devised an automated image analysis pipeline and constructed a predictive Random Forest model for spatial response assessment. Leveraging FET-PET and advanced MR imaging techniques, our model demonstrated a classification accuracy of 80,0% and exhibited promising AUCs of 0.875 through receiver operating characteristics (ROC) curve analysis.

Conventional MRI’s limitation in differentiating TP and PsP has spurred numerous investigations into advanced imaging modalities [4]. The rationale lies in the belief that these modalities, by visualizing key oncogenic processes like proliferation or angiogenesis, offer deeper insights into tumor biology. Indeed, studies have consistently shown superior results with the combined use of advanced imaging modalities compared to single-modality approaches [19]. ^,^ [34]^,^ [35] For instance, a recent study developed a classifier based on static FET-PET radiomics features that enables the differentiation between TP and PsP in pretreated gliomas with high diagnostic accuracy [14]. The feature importance analysis of our model revealed a significant contribution of FET-PET, which supports earlier findings in the literature on the high importance of FET-PET in this situation [16]. ^,^ [5] For example, a study conducted by Galldiks et al. demonstrated that both static and dynamic FET-PET uptake provide valuable information for distinguishing PsP from TP in glioblastoma patients [36]. While they reported no synergistic benefit of the combined assessment of static and dynamic FET-PET uptake, integrating dynamic PET information into the elucidation of spatial response heterogeneity is an interesting research direction.

In a recent work, Steidl et al. reported the sequential use of DSC perfusion and FET-PET in glioma response assessment [16]. In their model, patients with a very high rCBV > 2.85 were determined to have progressive disease (again on a global level), while for the remaining ambiguous patients, FET-PET was used. However, we want to emphasize that our Random Forest model relies on all three input sequences (FET-PET, CBV, contrast-enhanced T1w) jointly to discriminate different subparts of the suspected progress. Notably, lower perfusion values observed in treatment-related changes versus recurrent tumors, as highlighted by Prager et al., underscore the utility of DSC in discriminating between these entities, a finding reaffirmed in our study [34]. 

A common limitation in existing studies is the binary separation between TP and PsP, disregarding the complex intratumoral heterogeneity [5]. This complexity must be addressed for effective longitudinal treatment monitoring. In clinical practice, surgical biopsy remains the gold standard for diagnosing recurrent or residual disease [37]. However, its invasiveness and potential inaccuracies, such as undersampling, emphasize the need for non-invasive alternatives. Jiang et al. demonstrated the effective integration of advanced imaging into stereotactic biopsy planning [12]. The authors investigated the correlation between radiographic and histopathologic findings using volumetric amide proton transfer-weighted (APTw) image-directed stereotactic biopsy. Their findings indicate that APTw imaging can identify active tumor areas in heterogeneous brain lesions.

Both mRANO and RANO 2.0 criteria emphasize the importance of confirmatory scans for longitudinal response assessment [20]. ^,^ [38] Coupled with computational image analysis workflows, which amalgamate automated segmentation and registration for localized evaluation of tumor growth, this approach promises to unveil spatial heterogeneity in tumor response, as demonstrated in our proof-of-concept study. Notably, our pipeline facilitates fully automated image analysis, integrating automatic tumor segmentation, objective supervoxel clustering, feature extraction, and subsequent Random Forest classification without manual intervention.

Our intention to automate the analysis workflow also motivated our choice of supervoxels to form the basis for feature extraction and modeling. Supervoxels cluster neighboring voxels into semantically similar areas and, therefore, enable a fine-grained, regional analysis. Consequently, they have been used extensively in medical image analysis [39]. ^,^ [40] Unlike alternative approaches such as connected components, supervoxels ensure evenly spaced regions, preserve semantic information, and account for regional heterogeneity within connected components.

Given our dataset’s characteristics, we opted for the Random Forest classifier due to its ability to learn reliable classifiers even with smaller datasets [31]. More advanced deep learning strategies, on the other hand, typically require more significant amounts of data to perform effectively [41]. ^,^ [42] Another advantage of Random Forests over deep learning models is their inherent explainability in the form of feature importance. This attribute enables understanding of the classifier’s decisions, offering valuable insights into the underlying data, such as the pivotal role of high FET-PET signal.

### Limitations

Although this study provides valuable insights, there are several limitations that must be considered when interpreting the results. First, the study was conducted retrospectively at a single center, which may limit the generalizability of the results.

In addition, the utilization of only two scanners may limit the diversity of the data, potentially affecting the robustness of the conclusions across different imaging technologies or settings.

To ensure a homogeneous patient population and to minimize variability in measured parameters that may arise from different biological tumor characteristics, we selectively included only patients with *IDH* wild-type glioblastoma, resulting in a relatively small sample size. Further, five patients were included at two different time points. Although this may introduce potential bias into our model, it also provides a more accurate and representative snapshot of the “real world” clinical environment. Further, we ensured during tenfold cross-validation that all supervoxels from one patient were always represented in the same split to avoid information leakage.

We employed BraTS.Toolkit for automated image processing and tumor segmentation. Despite previous investigations into the capabilities of the pipeline and manual correction of all segmentations when necessary, there is still a possibility of slight variation in the segmentations that could have affected our results.

This is particularly relevant during the manual inspection and categorization of each lesion based on the changes observed in the follow-up images.

At present, each supervoxel is assessed individually. However, this is probably suboptimal, as a supervoxel’s neighborhood might provide relevant information for the classification. Similar to established tissue segmentation frameworks such as ANTs Atropos, it is well conceivable that implementation of “neighborhood-awareness”, for example, in the form of Markov Random Fields or Graph Networks, could further improve these response assessment models [26]. 

## Conclusion

To address the spatial heterogeneity in glioblastoma response, we introduce a novel approach utilizing a random forest classifier based on multimodal imaging and automated image analysis. This innovative method effectively distinguishes progressive tumor subparts from those showing similar appearances but remaining stable (or even decreasing) in the follow-up scan, indicative of pseudoprogression. Our findings underscore the significance of FET-PET features in this classification, which is consistent with previous research, but also highlight the value of multiparametric imaging. Notably, our study represents one of the first attempts to propose a predictive tool for spatially differentiated tumor response assessment in glioblastoma. While further validation on a larger scale is warranted, this approach holds immense promise for enabling more precise response evaluation in glioblastoma patients, potentially facilitating more personalized and localized treatment strategies in the future.

## Electronic supplementary material

Below is the link to the electronic supplementary material.

Supplementary Material 1

Supplementary Material 2

## Data Availability

Patient data (MR and PET images) are not publicly available due to data privacy reasons.
