# Supplementary material for: Resolving spatial response heterogeneity in glioblastoma
Source: Eur J Nucl Med Mol Imaging. 2024 Jun 5;51(12):3685–95. doi: 10.1007/s00259-024-06782-y (PMC11445274; doi:10.1007/s00259-024-06782-y)
Supplement: Supplementary file 1 — Supplementary Material 1 [file 259_2024_6782_MOESM1_ESM.pdf]

Titel: Resolving Spatial Response Heterogeneity in Glioblastoma

Journal: European Journal of Nuclear Medicine and Molecular Imaging

Julian Ziegenfeuter1, Claire Delbridge2, Denise Bernhardt3, Jens Gempt4,5, Friederike Schmidt-Graf6, Dennis Hedderich1, Michael Griessmair1, Marie Thomas1, Hanno S Meyer4,5, Claus Zimmer1, Bernhard Meyer4, Stephanie E Combs3, Igor Yakushev7, Marie-Christin Metz1,*, Benedikt Wiestler1,8,*

1. Department of Neuroradiology, School of Medicine and Health, Technical University of Munich, 81675 München, Germany
2. Department of Pathology, Technical University of Munich, 81675 München, Germany
3. Department of Radiation Oncology, School of Medicine and Health, Technical University of Munich, 81675 München, Germany
4. Department of Neurosurgery, School of Medicine and Health, Technical University of Munich, 81675 München, Germany
5. Department of Neurosurgery, University Medical Center Hamburg-Eppendorf, 20251 Hamburg, Germany
6. Department of Neurology, School of Medicine and Health, Technical University of Munich, 81675 München, Germany
7. Department of Nuclear Medicine, School of Medicine and Health, Technical University of Munich, 81675 München, Germany
8. TranslaTUM, Technical University of Munich, 81675 München, Germany * MM and BW contributed equally as senior authors

corresponding author: julian@ziegenfeuter.de

Supplementary Table 1: Hold-out test data AUC for the 10 individual cross-validation runs

Fold	Macro-weighted AUC
1	8.887.188.818.129.180
2	8.380.613.679.596.390
3	9.031.511.041.270.360
4	8.689.521.131.420.060
5	8.964.105.533.041.100
6	8.655.198.081.217.430
7	8.842.527.499.793.230
8	853.606.612.122.224
9	8.834.696.261.682.240
10	8.689.919.893.190.920
